# Slow expansion of multiple sclerosis iron rim lesions: pathology and 7 T magnetic resonance imaging

**DOI:** 10.1007/s00401-016-1636-z

**Published:** 2016-10-27

**Authors:** Assunta Dal-Bianco, Günther Grabner, Claudia Kronnerwetter, Michael Weber, Romana Höftberger, Thomas Berger, Eduard Auff, Fritz Leutmezer, Siegfried Trattnig, Hans Lassmann, Francesca Bagnato, Simon Hametner

**Affiliations:** 1 Department of Neurology, Medical University of Vienna, Vienna, Austria; 2 Department of Health Sciences and Social Work, Carinthia University of Applied Sciences, Klagenfurt, Austria; 3 Department of Biomedical Imaging and Image-guided Therapy, High Field Magnetic Resonance Centre, Vienna, Austria; 4 Institute of Neurology, Medical University of Vienna, Vienna, Austria; 5 Clinical Department of Neurology, Medical University of Innsbruck, Innsbruck, Austria; 6 Center for Brain Research, Medical University of Vienna, Vienna, Austria; 7 Neuroimmunology Division/Neuroimaging Unit, Department of Neurology, Vanderbilt University Medical Center, Nashville, TN USA

**Keywords:** Multiple sclerosis, Iron rim, Phase, 7 T MRI, SWI

## Abstract

In multiple sclerosis (MS), iron accumulates inside activated microglia/macrophages at edges of some chronic demyelinated lesions, forming rims. In susceptibility-based magnetic resonance imaging at 7 T, iron-laden microglia/macrophages induce a rim of decreased signal at lesion edges and have been associated with slowly expanding lesions. We aimed to determine (1) what lesion types and stages are associated with iron accumulation at their edges, (2) what cells at the lesion edges accumulate iron and what is their activation status, (3) how reliably can iron accumulation at the lesion edge be detected by 7 T magnetic resonance imaging (MRI), and (4) if lesions with rims enlarge over time in vivo, when compared to lesions without rims. Double-hemispheric brain sections of 28 MS cases were stained for iron, myelin, and microglia/macrophages. Prior to histology, 4 of these 28 cases were imaged at 7 T using post-mortem susceptibility-weighted imaging. In vivo, seven MS patients underwent annual neurological examinations and 7 T MRI for 3.5 years, using a fluid attenuated inversion recovery/susceptibility-weighted imaging fusion sequence. Pathologically, we found iron rims around slowly expanding and some inactive lesions but hardly around remyelinated shadow plaques. Iron in rims was mainly present in microglia/macrophages with a pro-inflammatory activation status, but only very rarely in astrocytes. Histological validation of post-mortem susceptibility-weighted imaging revealed a quantitative threshold of iron-laden microglia when a rim was visible. Slowly expanding lesions significantly exceeded this threshold, when compared with inactive lesions (*p* = 0.003). We show for the first time that rim lesions significantly expanded in vivo after 3.5 years, compared to lesions without rims (*p* = 0.003). Thus, slow expansion of MS lesions with rims, which reflects chronic lesion activity, may, in the future, become an MRI marker for disease activity in MS.

**Electronic supplementary material:**

The online version of this article (doi:10.1007/s00401-016-1636-z) contains supplementary material, which is available to authorized users.

## Introduction

Multiple sclerosis (MS) is a chronic disease of the central nervous system (CNS) associated with focal inflammatory demyelinating lesions in the white and grey matter [14]. Some lesions remyelinate early after the demyelinating event [16] and evolve into remyelinated shadow plaques, which protect against axonal degeneration [23]. Other lesions remain chronically demyelinated. Chronic demyelination fosters persistent low-degree neurodegeneration in the form of axonal transections [24]. A subset of lesions with inactive demyelinated centers maintains continuous myelin breakdown at the edge, which has led to the pathological concept of the slowly expanding lesion [36]. Pathologically, the edge of slowly expanding lesions is featured by a rim of activated microglia/macrophages harboring occasional myelin degradation products [15], few T cells [14, 36], and a considerable amount of axonal transections [15].

MS typically starts with relapsing-remitting course which progresses into secondary progression in 70% of patients. About 10% of patients begin the disease with a primary progressive course [32]. The common clinical feature of progressive MS is a continuous neurological decline in the absence of new and contrast-enhancing lesions and clinical relapses. There is currently no approved treatment to reduce disability accrual in progressive MS, which is partly due to our incomplete understanding of the pathobiological mechanisms underlying progression [32]. In a pathological survey on 2,476 WM plaques, slowly expanding lesions were predominantly found in progressive MS [15] and were suggested to indicate progressive disease activity [15, 36]. Focal T-cell-mediated CNS inflammation [4] causing relapses seems to differ from the typical microglia-mediated wide-spread inflammation that features progressive MS [26]. Mitochondrial DNA deletions, oxidative stress [20], and iron accumulation and its liberation during demyelination [21] are thought to be key factors of neurodegeneration in progressive MS [28].

Iron accumulation has been described within microglia/macrophages at the edges of slowly expanding [5, 21] and some inactive lesions, but was not observed around shadow plaques [21]. Questions remain whether iron accumulation surrounds other lesion types, whether it differs between slowly expanding and inactive lesions, and whether it is, indeed, absent from edges of shadow plaques. Based on proper pathological characterization, edge-related iron accumulation might, therefore, separate expanding non-remyelinating lesions [3] from those with increased remyelination probability, and ultimately become a useful imaging biomarker for disease activity in stages of MS, where contrast enhancement of lesions is rare or absent.

Magnetic resonance imaging (MRI) and post-mortem studies showed that edge-related iron accumulation is captured by a rim-shaped signal around chronic WM lesions when using phase [3, 7, 22, 30, 39], susceptibility-weighted imaging (SWI) [18], multi-echo gradient echo *R* ^2*^ [40], or quantitative susceptibility mapping (QSM) [10, 12]. These rims were seen in patients with either relapsing-remitting or secondary progressive MS [30, 40], but were absent from supratentorial neuromyelitis optica (NMO) lesions, which render iron rims potentially helpful for distinction between MS and NMO lesions [10]. Longitudinal analyses of persistent rim lesions in MS thus far showed lack of expansion over 2.5 years [7] or slight shrinkage within the first 12 months after gadolinium enhancement resolution [3], challenging the notion that iron rims surround slowly expanding lesions in vivo.

To gain knowledge on the pathological and in vivo features of rim lesions, we examined the association between rim-shaped iron accumulation at the edges and the pathological stages of lesions in a sample of 28 post-mortem MS cases. We then examined whether lesions encircled by a rim-shaped signal in SWI are more likely to expand over a period of 3.5 years than those without rims in a prospective longitudinal study in seven patients with MS using a fluid attenuated inversion recovery/SWI fusion sequence (FLAIR–SWI) at 7 Tesla (7 T) [2, 11, 17].

## Materials and methods

### Study design, samples and patients

This study is a collaborative project between the Medical University in Vienna and the Neurology Department of Vanderbilt University, Nashville, TN.

**Fig. 1:**
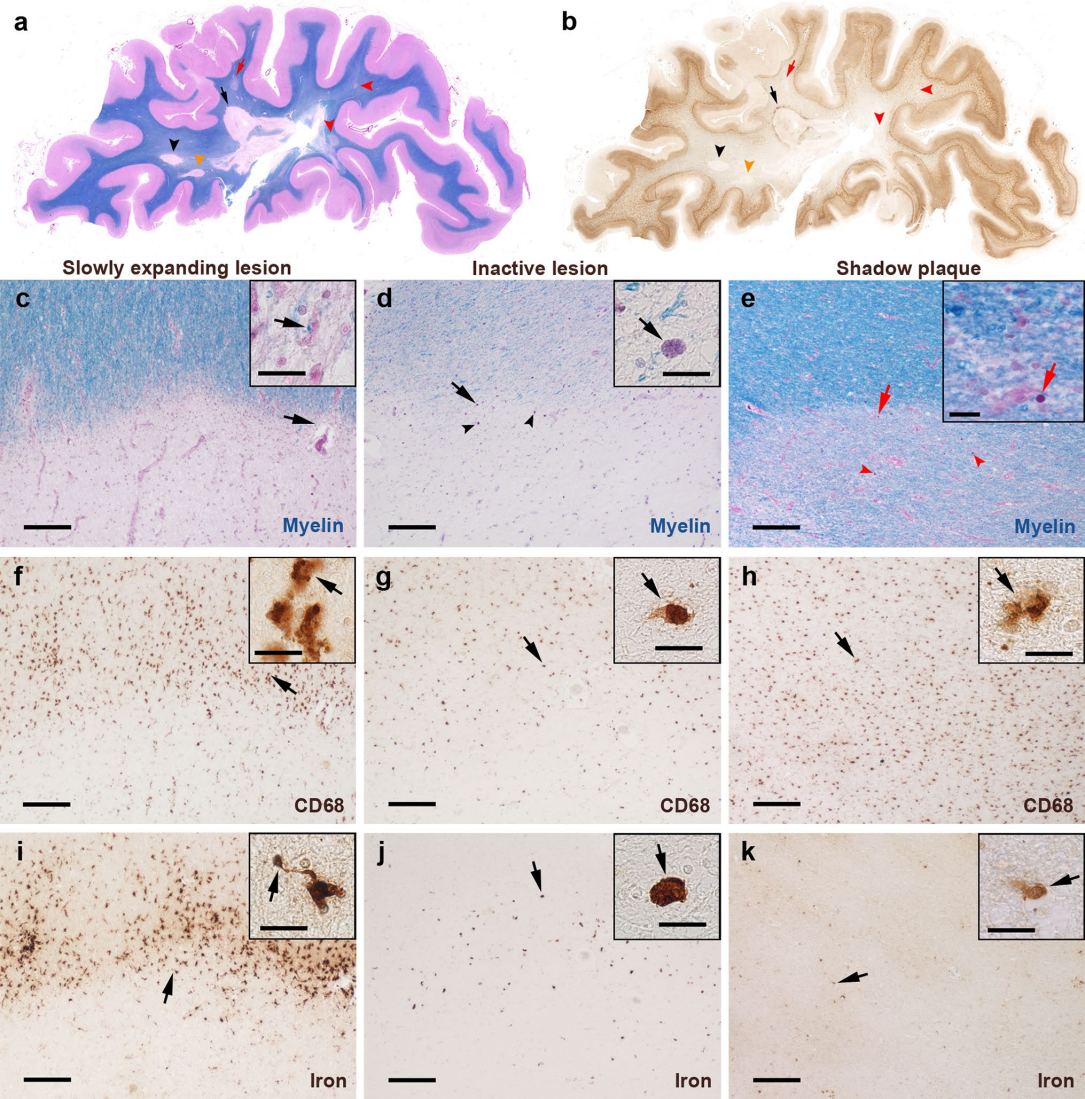
Iron-related pathology of slowly expanding and inactive lesions as well as shadow plaques. Rim-like iron accumulation was observed around a subset of demyelinated WM lesions but hardly around shadow plaques. **a**, **b** Hemispheric sections of the temporal lobe of SPMS case 13, stained for myelin (**a**, *blue*) and iron (**b**, *brown*), show several WM lesions. *Black arrows/arrowheads* indicate slowly expanding lesions, *red arrows/arrowheads* indicate shadow plaques, and *orange arrowheads* indicate an inactive lesion. One slowly expanding iron rim lesion is indicated by a *black arrow*, magnified in **c**, **f,** and **i**. *Red arrow* indicates the edge of a shadow plaque, magnified in **e**, **h,** and **k**. The micrographs depict a slowly expanding lesion from SPMS case 13, an inactive lesion from SPMS case 18, and a shadow plaque from case 13. **c**–**k** The horizontal lesion edges divide micrographs into myelinated WM (*top half*) and lesion (*bottom half*). **c** Slowly expanding edge is characterized by intracellular LFB-positive myelin degradation products (*arrows*, *inset*). **f** Microglia/macrophages at the edge display activated morphology (*arrows*, *inset*) and are reduced in the center. **i** Iron rim is formed by iron-laden microglia/macrophages, which frequently shows dystrophic morphology, such as process swellings and buddings (*arrows*, *inset*). **d** This inactive edge contains macrophages with intracellular lipofuscin lipids (*arrows*, *inset*, *arrowheads*), suggesting remote demyelinating activity of the lesion. **g** Fewer microglia/macrophages (*arrows*, *inset*) at the edge, when compared with **f**, and loss of microglia/macrophages in the center. **j** Few iron-laden microglia/macrophages at this inactive edge, while iron content in individual macrophages (*arrows*, *inset*) is comparable to levels observed at slowly expanding edges. **e** No lipid-laden macrophages but corpora amylacea (*red arrows*, *inset*, *red arrowheads*) are found at this shadow plaque edge. **h** Maintained microglia/macrophage density (*arrows*, *inset*) across the shadow plaque edge and center, which contrasts microglia loss in chronically demyelinated centers. **k** No edge-contouring iron accumulation around this shadow plaque. Low iron content is observed in few cells (*arrows*, *inset*).* Scale bars* 200 µm;* inset scale bars* 20 µm

**Fig. 2:**
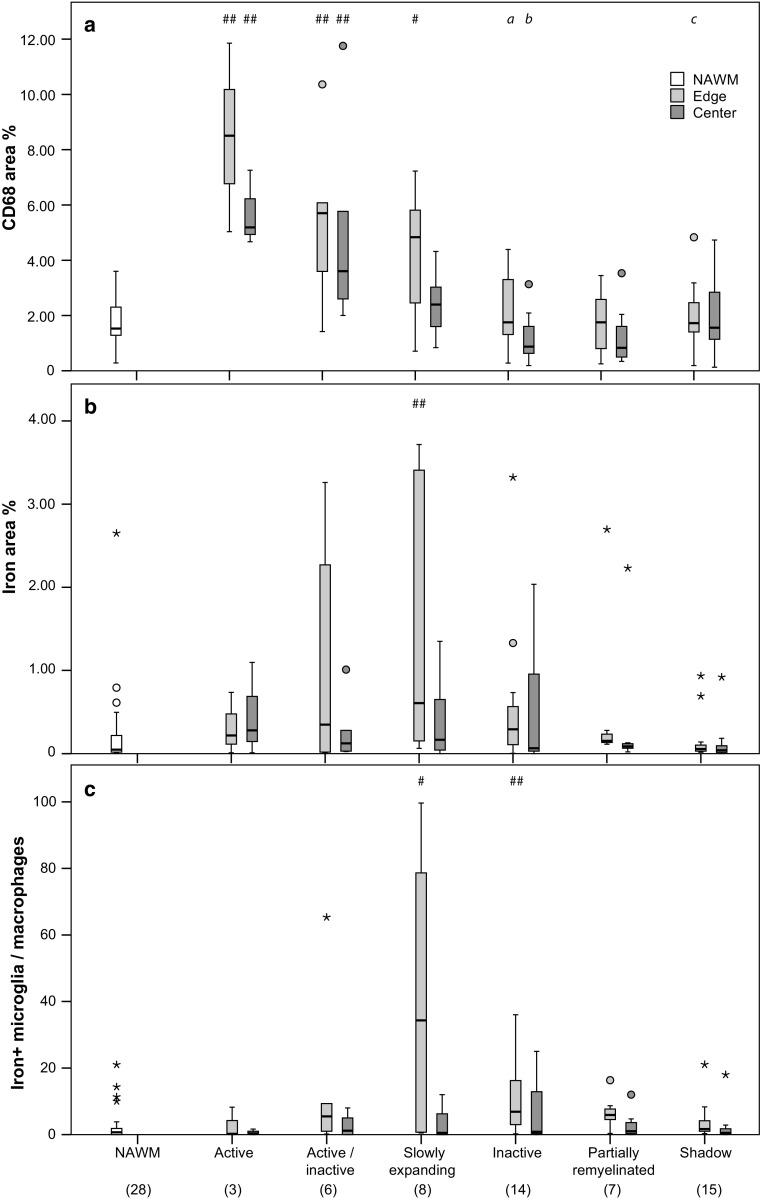
Quantitative pathological data of the first sample. Optical densities (area fraction) of CD68^+^ microglia/macrophages (**a**) and total non-heme iron (**b**) as well as manually counted iron-laden cells with microglia or macrophage morphology (**c**) within an area of 0.43 mm^2^. Hashes indicate significant differences compared with NAWM (#: *p* < 0.05, ##: *p* < 0.01). Data represent case-based averages of different lesion types. Numbers in brackets at the bottom indicate numbers of multiple sclerosis cases and data points contributing to the boxplots and statistical tests

**Fig. 3:**
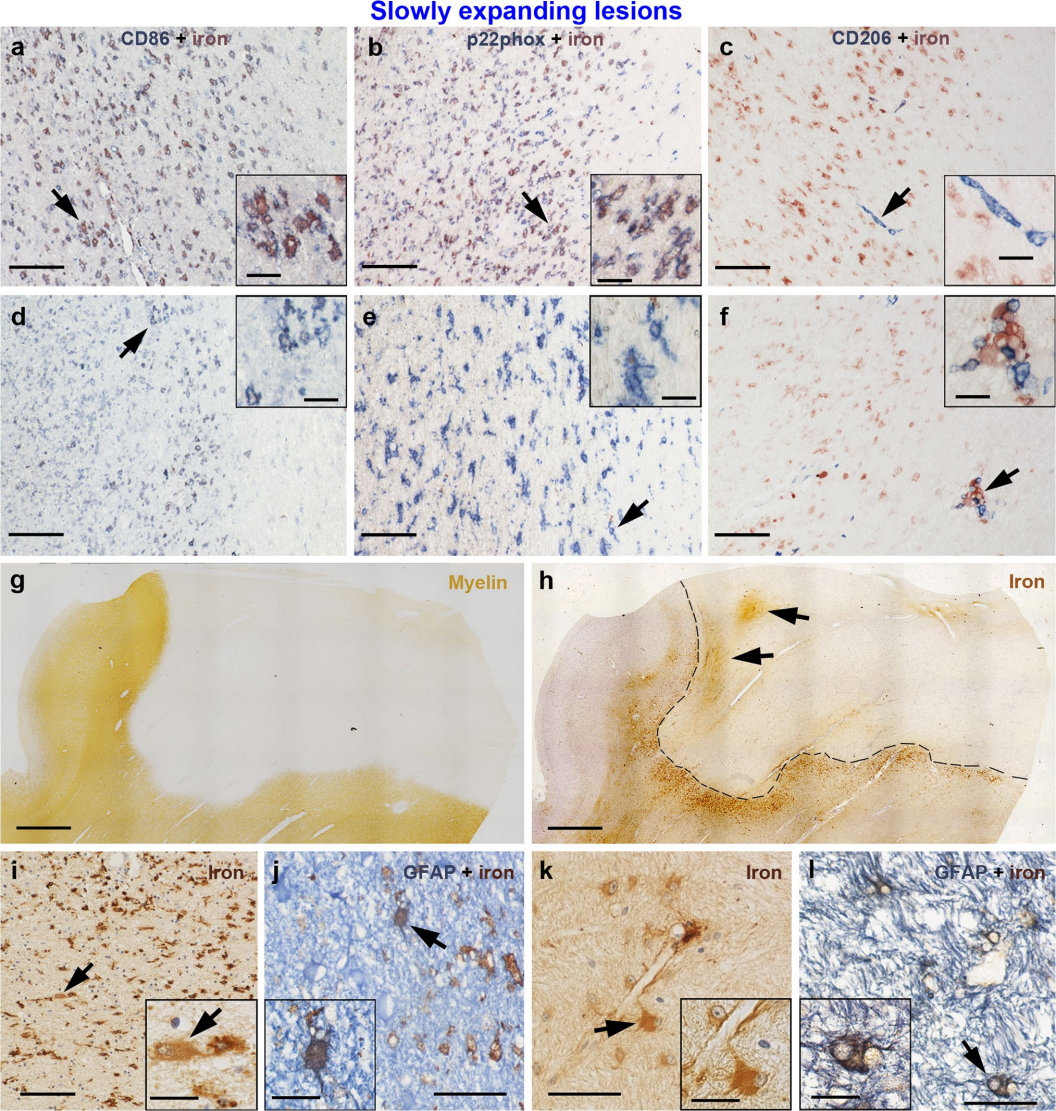
Activation status of iron-laden microglia/macrophages and presence of iron-laden astrocytes in slowly expanding lesions of the second sample. (**a, b**) Many microglia/macrophages are iron-laden in this slowly expanding iron rim region, and virtually all of them express the pro-inflammatory markers CD86 (**a**) and p22phox (**b**). (**d, e**) In another region of the same rim with few iron-laden cells, still many microglia/macrophages express CD86 (**d**) and p22phox (**e**). (**c**) Only few microglia/macrophages were anti-inflammatory and CD206-positive. Most of them were located in perivascular spaces, and only a minority of CD206-positive cells was iron-laden (**f**). (**g**) Slowly expanding lesion is visible in the myelin (PLP) staining with a sharply demarcated border. (**h**) Adjacent section stained for iron. The border of this iron rim lesion is outlined by a dashed black line. Two black arrows indicate focal perivascular iron accumulation within the lesion core. The region indicated by the upper arrow is magnified in **k** and **l**. (**i**) Iron-laden astrocytes in the iron rim were sparse (arrows, inset) and showed weaker iron reactivity, when compared with surrounding microglia/macrophages. (**j**) Rare astrocytic iron accumulation in the rim was confirmed with double-labeling with GFAP (astrocyte marker). (**k**) Astrocyte (arrow, inset) with contact to a vessel wall shows moderate iron accumulation. Many iron-laden astrocytes are observed in this region of perivascular iron accumulation within the lesion. (**l**) Confirmation of astrocytic iron with double-labeling. The same region as **k**. *Scale bars* 100 µm (**a**–**f**, **i**), 2 mm (**g**, **h**), 50 µm (**j**–**l**); *inset scale bars* 20 µm

**Fig. 4:**
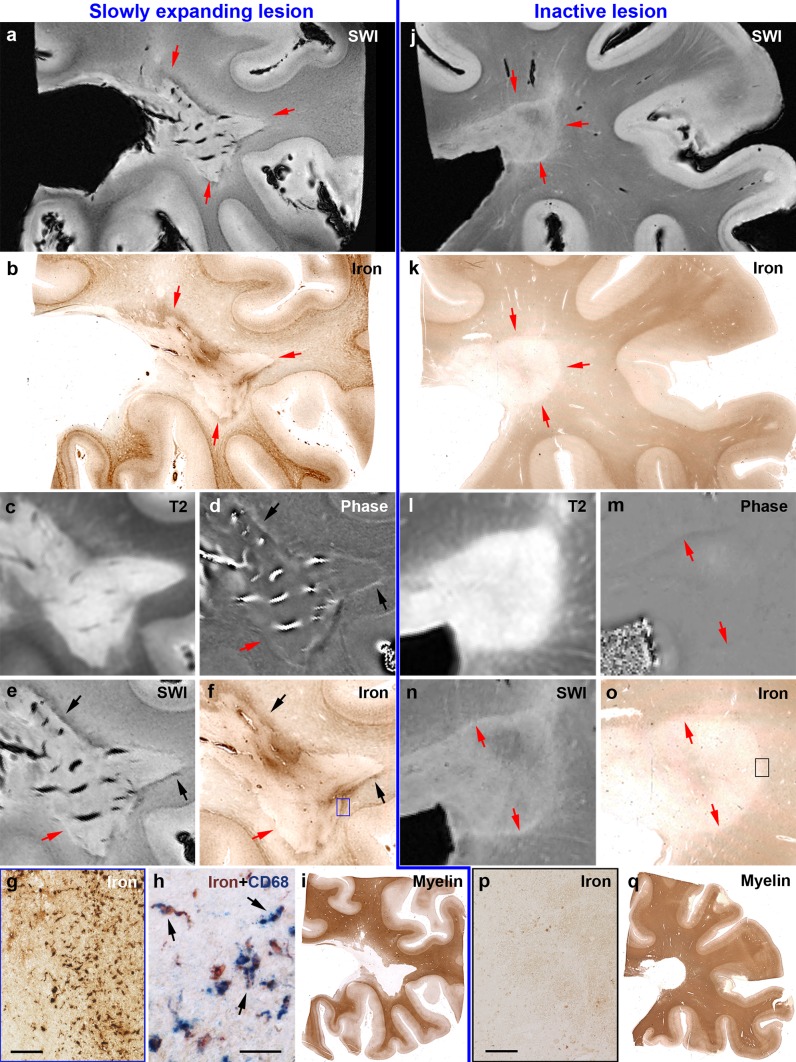
Post-mortem validation of SWI and underlying phase images at 7 T for the sensitive and specific detection of iron rims around MS lesions. *Left* **a** Slowly expanding rim lesion (*red arrows*) of PPMS case 26 with hypointense portions along the lesion edge around a hyperintense lesion center. **b** Corresponding iron staining confirms iron accumulation at the lesion edge (*red arrows*). **c** Extent of the hyperintense lesion in the *T*2 image. *Black arrows* in (**d**, **e**, **f**) highlight portions of edge-related hyperintense phase and hypointense SWI, which correspond to iron accumulation. *Red arrows* in (**d**, **e**, **f**) highlight portions which lack hyperintense phase, hypointense SWI and iron accumulation. **f** The *blue rectangle* is magnified in (**g**) and shows iron-laden microglia/macrophages, which is confirmed by double-labeling with the microglia/macrophage marker CD68 (**h**). *Arrows* indicate double-labelled microglia/macrophages. **i** Extent of the demyelinated lesion in PLP staining for myelin. *Right* **j** inactive lesion (*red arrows* in **j**, **k**) of PPMS case 27 without edge-related hypointensities around the hyperintense lesion center. **k** Corresponding iron staining confirms lack of edge-related iron accumulation. **l** Extent of the hyperintense lesion in the *T*2 image. *Red arrows* in (**m**, **n**, **o**) indicate lesion edge devoid of hyperintense phase, hypointense SWI, and iron accumulation. **o** *Black rectangle* is magnified in (**p**), showing the absence of iron-loaded microglia/macrophages. **q** Extent of the demyelinated lesion in PLP staining for myelin. *Scale bars* = 100 µm (**g**, **p**) and 30 µm (**h**)

**Fig. 5:**
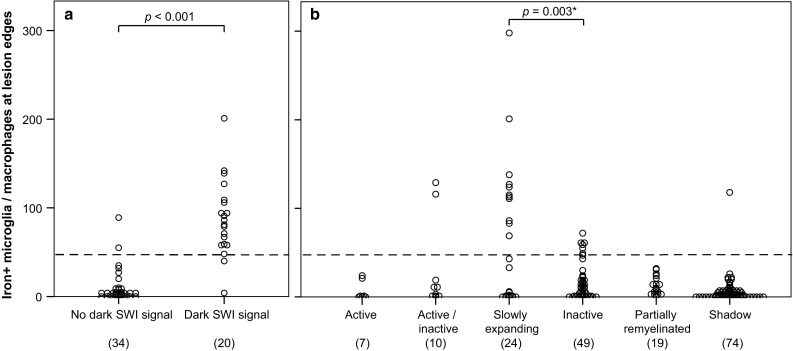
Iron-laden microglia/macrophages at edges of WM lesions of the first sample. **a** Establishment of a post-mortem threshold (*dashed line*) for manually counted iron-laden microglia/macrophages to induce a* dark* *rim* signal in SWI, based on four multiple sclerosis cases and four WM lesions with available post-mortem scans. Each data point represents one ROI. Numbers in brackets indicate numbers of ROIs. **b** Application of the threshold (*dashed line*) for the edges of all 183 WM lesions indicates that 2/10 active/inactive, 11/24 slowly expanding, 6/49 inactive lesions, and 1/74 shadow plaques exceeded this threshold. *P value of Chi-square test between slowly expanding and inactive lesion edges which were either below or above the threshold. Each data point represents the value of one lesion edge. Numbers in brackets indicate numbers of data points and individual lesions

The post-mortem study was performed on two partly overlapping samples of 32 MS cases in total, denoted as first and second samples. The first sample (28 cases) served for global characterization of MS WM lesions, their activity state, CD68 expression (microglia/macrophages), and iron accumulation (Figs. 1, 2, 4, 5). It consisted of double-hemispheric or large sections. Thirteen cases were provided by the University of Vanderbilt which received them from the MS Society Tissue Bank (*n* = 12) and the Rocky Mountain MS Center, Colorado, USA (*n* = 1). Two cases were collected at the Institute of Neurology, Medical University of Vienna, Austria, and thirteen were from the archive at the Center for Brain Research, Medical University of Vienna, Austria. For the first sample, clinical data and numbers of WM lesions per case are provided in Table 1. The second sample (10 cases) served for the characterization of the inflammatory activation status of iron-laden microglia/macrophages and iron accumulation in astrocytes within iron rims (Fig. 3, supplementary Fig. 1). The second sample consisted of 10 cases, 6 cases from the first sample, and 4 additional cases from the archive at the Center for Brain Research, Vienna (Table 2).

**Table 1:** Demographics and clinical characteristics of the first sample of pathological MS cases

Case ID	Age at death (years)	Sex (f/m)	Disease duration (years)	Disease course	Lesion characterization	Analyzed/iron rim lesions: n/n (%)
1	35	m	0.13	AMS	3 A	3/0 (0)
2	40	f	10	RRMS	3 A	3/0 (0)
3	60	f	41	RRMS	1 PR	1/0 (0)
4	69	m	43	RRMS	1 SP	1/0 (0)
5	31	m	11	SPMS	1 A, 2 A/I, 1 SP	4/0 (0)
6^a^	34	m	10	SPMS	2 A/I, 7 SEL, 4 SP	13/9 (69)
7	43	m	16	SPMS	5 SEL, 3 I	8/3 (38)
8	45	f	20	SPMS	2 SEL, 6 I, 1PR, 3 SP	12/0 (0)
9	46	f	22	SPMS	1 A/I, 1 SEL, 9 I, 1 PR	12/1 (8)
10^a^	46	f	37	SPMS	2 SEL, 3 I, 10 PR, 17 SP	32/4 (12)
11	55	m	31	SPMS	13 SP	13/0 (0)
12	56	m	31	SPMS	3 A/I, 1 I	4/2 (50)
13	61	f	33	SPMS	2 SEL, 1 I, 2 SP	5/1 (20)
14	62	f	26	SPMS	1 A/I, 3 I, 2 SP	6/0 (0)
15	66	m	51	SPMS	2 PR, 4 SP	6/2 (33)
16	71	f	35	SPMS	2 I, 1 SP	3/1 (33)
17	71	m	46	SPMS	1 A/I, 1 I, 3 PR	5/1 (20)
18	72	f	33	SPMS	1 I	1/0 (0)
19	74	f	48	SPMS	7 I	7/5 (71)
20	79	f	48	SPMS	2 SP	2/0 (0)
21	83	f	47	SPMS	3 I	3/1 (33)
22	88	f	30	SPMS	1 SP	1/0 (0)
23	88	f	36	SPMS	1 PR, 1 SP	2/0 (0)
24	90	f	33	SPMS	No lesion	only NAWM
25	55	f	5	PPMS	3 I, 14 SP	17/1 (6)
26^a^	62	m	12	PPMS	2 SEL	2/1 (50)
27^a^	67	m	7.25	PPMS	3 SEL	3/1 (33)
28	n.a.	f	n.a.	n.a.	6 I, 8 SP	14/2 (14)
All	61.07 ± 16.77 (mean ± SD)	17/11 (f/m)	28.24 ± 14.75 (mean ± SD)	3/23 (RRMS/PMS)	7 A, 10 A/I, 24 SEL, 49 I, 19 PR, 74 SP	183/35 (19)

*A* active, *A/I* active/inactive, *AMS* acute MS, *f* female, *I* inactive, *m* male, *n.a.* not available, *NAWM* normal appearing white matter, *PMS* progressive MS, *PPMS* primary progressive MS, *PR* partially remyelinated, *RRMS* relapsing-remitting MS, *SD* standard deviation, *SEL* slowly expanding lesion, *SP* shadow plaque, *SPMS* secondary progressive MS, *yrs* years

^a^Case included for sequence validation

**Table 2:** Demographics and clinical characteristics of the second sample of pathological MS cases, used for characterization of iron-laden microglia/macrophages and astrocytes

Case ID	Age at death (yrs)	Sex (f/m)	Disease duration (years)	Disease course
6^a^	34	m	10	SPMS
10^a^	46	f	37	SPMS
12^a^	56	m	31	SPMS
13^a^	61	f	33	SPMS
26^a^	62	m	12	PPMS
27^a^	67	m	7.25	PPMS
29	41	m	11.4	SPMS
30	53	f	20.1	SPMS
31	76	m	31	SPMS
32	53	m	14	PPMS
All	54.90 ± 12.45 (mean ± SD)	3:7 (f:m)	20.68 ± 11.21 (mean ± SD)	10 PMS

*f* female, *m* male, *PMS* progressive MS, *PPMS* primary progressive MS, *SD* standard deviation, *SPMS* secondary progressive MS, *yrs* years

^a^Case also included in the MS sample (*n* = 28) shown in Table 1

For the in vivo study, ten patients with MS [35] were consecutively enrolled. Patients fulfilled the following inclusion criteria: age >18 years, expanded disability status scale (EDSS) ≤6.5 [25], no steroid therapy during the last 3 months, and no contraindication for 7 T MRI.

They underwent neurological examinations and brain MRIs at the following timepoints: study entry or baseline, years 1, 2, and 3.5. Patients’ demographic and clinical data at the time of the study entry are given in Table 3.

**Table 3:** Demographics and clinical characteristics of the patients included in the in vivo study

Patient	Age (yrs)^b^	Sex	Disease duration (yrs)^b^	Disease course	EDSS treatment				Total//discrete/rim lesions: n//n/n (% rim of discrete lesions)
					Baseline	1-yr FU	2-yr FU	3.5-yr FU	
1^a^	21	f	3	RRMS	0	0	1.0	n.a.	40//5/2 (40)
					No	No	GA	–	
2^a^	27	m	7	RRMS	1.5	3.0	2.5	2.5	23//8/6 (75)
					INF beta-1A	INF beta-1A	INF beta-1A	INF beta-1A	
3^a^	28	f	3	RRMS	1.0	1.0	1.0	1.0	14//6/2 (33)
					INF beta-1A	FTY	FTY	FTY	
4^a^	29	f	5	RRMS	2.0	2.0	2.0	2.5	29//16/11 (69)
					GA	GA	GA	GA	
5^a^	36	m	3.5	RRMS	2.0	1.5	2.0	2.5	24//5/2 (40)
					INF beta-1A	FTY	FTY	FTY	
6^a^	52	f	6	RRMS	3.0	4.5	5.5	5.5	37//7/3 (43)
					GA	NTZ	NTZ	NTZ	
7	52	f	25	SPMS	6.5	6.5	Excl.	Excl.	n.a.//n.a./n.a.
					INF beta-1A	INF beta-1A	–	–	
8^a^	53	m	28	SPMS	6.5	6.5	6.5	7.0	16//5/2 (40)
					No	No	No	No	
9	60	f	20	SPMS	6.5	Excl.	Excl.	Excl.	n.a.//n.a./n.a.
					INF beta-1b	–	–	–	
10	62	f	37	SPMS	6.0	6.0	6.0	6.5	34//0/0 (0)
					No	No	No	No	
	42 (±14.5) mean (±STD)	7/3 total numbers (f/m)	14 (±11.9) mean (±STD)	6/4 total numbers (RR/SP)	2.5 (0–6.5) median (range)	3 (0–6.5) median (range)	2.25 (1.0–6.5) median (range)	2.5 (1.0–7.0) median (range)	183//52/28 (53.8) total numbers (%)

*EDSS* expanded disability status scale, *excl.* excluded, *f* female, *FU* follow-up, *FTY* fingolimod, *GA* glatiramer acetate, *INF* interferon, *m* male, *n.a.* not applicable, *NTZ* natalizumab, *RRMS* relapsing-remitting multiple sclerosis, *SD* standard deviation, *SPMS* secondary progressive multiple sclerosis, *yrs* years, *%* percentage of discrete lesions that have a rim

^a^Patient contributed to longitudinal lesion volume data

^b^At timepoint of study initiation 2010

### Neuropathology

The first sample (Table 1) consisted of 31 formalin-fixed paraffin-embedded tissue blocks comprising double-hemispheric (*n* = 24) and large (*n* = 7, tissue size from 5 × 5 × 1 cm up to one hemisphere) blocks. For four cases (MS 2, 13, 26, 27), only large but no double-hemispheric blocks were available. For three cases (MS 6, 10, and 26), two blocks per case were included. The second sample consisted of ten blocks of ten cases. Three large blocks of the first sample were also included in the second sample and seven were additional routine blocks used for the second sample only. Consecutive double-hemispheric or large sections were cut with a tetrander microtome at a thickness of 10 µm and mounted on glass slides. Routine sections were cut at a thickness of 5 microns and mounted on glass slides. Sections were stained for hematoxylin and eosin to exclude confounding pathologies. Luxol fast blue-periodic acid Schiff (LFB-PAS) myelin staining was performed for assessment of WM lesions, including demyelinating activity and remyelination (Fig. 1a). DAB-enhanced Turnbull blue staining for di- and trivalent (total) non-heme iron was done as previously described [21, 29]. Immunohistochemistry for the myelin protein proteolipid protein (PLP) and the lysosomal glycoprotein CD68, indicating activated microglia/macrophages, was performed as described [21]. Sources, pretreatments and dilutions of primary antibodies are listed in Table 4. CD68 stainings were not counterstained to facilitate subsequent digital quantification. Double-labeling of iron with immunohistochemistry for the microglia/macrophage activation markers p22phox, CD86, CD206, and the astrocyte marker GFAP (Fig. 3) was performed on the second sample. Double-labeling of iron with CD68 (Fig. 4n) was performed on MS 26. For double-labelings, Turnbull blue staining was performed as described, but developed with aminoethyl carbazole (p22phox, CD86, CD206, and CD68) under microscopic control [21]. For double-labeling of iron with the astrocyte marker GFAP (Fig. 3), Turnbull staining was developed with DAB according to the protocol used for single labelings. After washing in aqua bidestillata, the sections were steamed for antigen retrieval (Table 4). Blocking of non-specific antibody binding was followed by incubation of the primary antibodies overnight at 4 °C. Appropriate secondary antibodies were applied for 1 h at room temperature. Secondary antibodies were either biotinylated (for p22phox, CD86, CD206, or GFAP) or directly conjugated to alkaline phosphatase (for CD68). If a biotinylated secondary antibody was used, sections were further incubated with avidin-conjugated alkaline phosphatase for 1 h at room temperature. The sections were developed with Fast Blue substrate (Sigma) at 37 °C.

**Table 4:** Primary antibodies used for immunohistochemistry

Target	Antibody type	Dilution	Antigen retrieval	Source (product number)	Protocol
PLP	Mc mouse	1:1000	60 min steaming with EDTA, pH 8.5	AbD Serotec, Oxford, UK (MCA839G)	SL
CD68	Mc mouse	1:100	60 min steaming with EDTA, pH 9	Dako, Carpinteria, CA (M0814)	SL, DL
CD86	Pc goat	1:50	60 min steaming with EDTA, pH 9	R&D Systems (AF-141-NA)	DL
CD206	Mc mouse	1:200	45 min steaming with EDTA, pH 9	Abcam (ab 117644)	DL
GFAP	Pc rabbit	1:3000	45 min steaming with EDTA, pH 8.5	Thermo Scientific, Waltham MA (MS-1376-P1)	DL

*CD* cluster of differentiation, *DL* double-labeling, *EDTA* ethylenediaminetetraacetic acid, *GFAP* glial fibrillary acidic protein, *Mc* monoclonal, *Pc* polyclonal, *PLP* proteolipid protein, *SL* single-labeling

### Histopathological lesion characterization

Characterization of WM lesions was performed as described [9, 15]. Active lesions harbored degradation products reactive for LFB and PLP inside macrophages [9] throughout the lesion. Active/inactive lesions showed a confined presence of macrophages with LFB- and PLP-reactive degradation products at the lesion edge (active zones). In this type of lesions, numerous predominantly perivascular macrophages containing PAS-positive lipids [27] were seen in the lesion cores, suggesting more remote demyelinating activity (inactive zones). Slowly expanding and inactive lesions showed sharply demarcated borders [15], inactive demyelinated centers without any degradation products [15], and occasional LFB-positive myelin degradation products in microglia or macrophages at edges of slowly expanding but not inactive lesions (Fig. 1). Remyelinated shadow plaques were identified by their sharply demarcated homogeneous reduction of LFB staining intensity [8] and were classified as shadow plaques if the whole lesional area was remyelinated. Partially remyelinated lesions showed areas of remyelination and complete demyelination [8].

### Histological quantification

Digital color (RGB) images sized 0.76 × 0.57 mm (0.43 mm^2^) were taken at constant illumination and microscope conditions [21] from different ROIs in the slides stained for iron and CD68. Three ROIs, at least 1 cm away from any MS lesion, represented the NAWM. A mean value across the three NAWM-ROIs was computed for the analyses. From each WM lesion, three images at the lesion edge and one in the lesion center were taken. For both iron and CD68 stainings, the three edge images were placed with regard to the most pronounced, least pronounced, and in-between CD68 edge expression, regardless of presence of iron. One center image was placed in the lesion center. Images were quantified using custom-written plugins for the ImageJ version 1.43r, which have been used before [21]. After grey-scale conversion of the images, the plugins apply a constant threshold after subtracting the mean grey value from the image, yielding a numerical estimation of CD68 and iron staining (area fraction) (Fig. 2a, b). On the RGB images of the iron stainings, cells with strong iron accumulation displaying microglia or macrophage morphology were manually counted (Fig. 2c). Values of the three edge images and data of multiple lesions of a lesion type per case were averaged to finally represent each lesion type per ROI per case with one data point for plotting and statistical testing (case-based averages) (Fig. 2). In the second sample, the p22phox-iron and CD206-iron double-labelings (Fig. 3) were quantified by manual counting of p22phox or CD206 single-positive cells, iron single-positive cells (on p22phox-iron stainings only), and double-positive cells (supplementary Fig. 2). Total p22phox or CD206-positive cells were determined by adding p22phox or CD206 single-positive cells to the double-labeled cells. For these quantifications, two ROIs were chosen in the edges of rim lesions: one ROI with the lowest, and one ROI with the highest numbers of iron-laden cells found in the whole lesion edge.

### Post-mortem MRI

#### Acquisition and post-processing

Prior to paraffin embedding, four large formalin-fixed tissue samples (MS 6, 10, 26, and 27) were imaged using a 7 T whole body MR system (Magnetom^®^ Siemens Healthcare, Erlangen, Germany) equipped with a 72 mm volume coil (RAPID^®^ Biomedical, Würzburg, Germany). Samples were placed in a cylindrical, custom-fabricated polyvinyl chloride container and immersed in perfluorinated fluid (Galden^®^ SV 80, Solvay Specialty Polymers, Milan, Italy). A three-dimensional (3D) SWI sequence was obtained using the following parameters: echo time (TE) = 15 ms, repetition time (TR) = 24 ms, in plane image matrix = 576 × 576 pixels, resolution = 0.14 × 0.14 × 0.35 mm, slices = 120–144 (sample-dependent), acquisition time (one measurement) = 27:39–33:18 min, and averages = 6. Phase images were filtered using Homodyne filtering [31]. SWI images were created by applying four multiplications of a positive phase mask to the SWI magnitude data [17, 19]. *T*2-weighted data were acquired using a two-dimensional (2D) turbo spin echo (TSE) sequence with TE = 34 ms, TR = 5370 ms, resolution = 0.11 × 0.11 × 0.6 mm, 60 slices, acquisition time (one measurement) = 10:50 min, and averages = 5.

#### Lesion identification and analysis

WM lesions were identified on *T*2-weighted TSE and SWI images (Fig. 4). A threshold for the number of iron-laden microglia/macrophages inducing a dark SWI rim was determined by taking 10 to 16 images of iron stainings per lesion along the edges (54 ROIs in total) and counting of iron-laden microglia/macrophages. On the matched SWI scans, the presence or absence of a dark edge-related SWI signal was outlined to determine whether a pathological ROI corresponded to a hypointense SWI signal or not. This evaluation was performed in a blinded manner by two different investigators. The threshold was set to have equal numbers of data points (i.e., 2) above the threshold in “no dark SWI signal” ROIs and below it in “dark SWI signal” ROIs (Fig. 5a). Microglia/macrophage countings in all lesion edges were plotted together with this threshold (Fig. 5b). Slowly expanding and inactive lesion edge data were dichotomized using this threshold for Chi-square analysis. Here, only one edge image of the iron staining per lesion corresponding to the maximum CD68 expression as well as single lesion data (no case-based averages) were plotted and tested.

### In vivo MRI

#### Acquisition and post-processing

Imaging was performed on the same 7 T MR system, using a 24-channel coil (Nova Medical, Wilmington, USA) first and a 32-channel radio frequency (RF) coil (Nova Medical, Wilmington, USA), as it became available. FLAIR images were acquired using a TSE sequence with variable flip-angle echo trains. Detailed information on pulse sequence parameters has been published previously [11]. SWI data were acquired using a 3D fully first-order flow-compensated SWI sequence with TE = 25 ms, TR = 38 ms, image matrix = 704 × 704, slices = 96, parallel imaging factor = 2, acquisition time = 13:56 min, and resolution = 0.3 × 0.3 × 1.2 mm. Phase filtering and SWI image processing was performed by the manufacture. The FLAIR sequence was then combined with the filtered SWI phase data (vendor-provided) as described previously, referred to as FLAIR–SWI contrast [17].

*T*1-weighted data were acquired using an magnetization-prepared rapid gradient echo (MP-RAGE) sequence with the following parameters: TR/inversion time (TI)/TE = 3800/1700/3.55 ms, image matrix = 320 × 320; resolution = 0.75 × 0.72 × 0.7 mm; slices = 208; and parallel imaging factor = 2; acquisition time = 10:29 min. Post-contrast images (acquired only at the first year of follow-up) were obtained 10 min after the injection of the contrast agent gadobenate dimeglumine (Multihance^®^, Bracco Imaging S.P.A., Colleretto, Italy), administered at the dose of 0.2 ml/kg body weight.

#### Lesion identification and quantification

Lesions detected in the frontal, parietal, and occipital lobes as well as the superior parts of the temporal lobes of the supratentorial brain were included in the analysis. Rim and non-rim WM lesions were identified at baseline and lesion volumes quantified annually on the FLAIR–SWI images by a neurologist specialized in MS (ADB), who was supervised by a certified radiologist (ST). Rim lesions were defined as discrete hyperintense WM lesions in FLAIR images entirely or partially surrounded by a rim of decreased signal on FLAIR–SWI. The rim was required to be seen on at least three contiguous slices. We also identified discrete hyperintense lesions without a rim at their edges and separated them from confluent WM lesions without a rim. Rim lesions and discrete but not confluent non-rim lesions were manually traced on the FLAIR–SWI images and volumes computed using *Display*, part of the MINC toolbox (http://packages.bic.mni.mcgill.ca). Fifteen randomly selected rim or non-rim lesion volumes were segmented twice to determine the intraclass correlation (ICC) of the volume measurements.

### Statistical analysis

Data were analyzed using IBM SPSS^®^ Statistics for Windows Version 20. Normally distributed metric data are described using mean ± standard deviation (SD). In case of skewed (histological) data, boxplots indicating medians, interquartile ranges (IQR), 1.5 × IQR (whiskers), and outliers (circles and stars) are presented. Nominal data are described using absolute frequencies and percentages. Due to non-normal distribution of histological data, conservative non-parametric Kruskal–Wallis and post-hoc Mann–Whitney *U* tests were used for comparison of regions of interest and corrected for multiple comparisons according to Bonferroni–Holm. To test the association between categorical variables (i.e., above/below threshold and slowly expanding/inactive lesions), a Chi-square test was performed. A mixed-model ANOVA (fixed within-subject factor ‘time’, fixed between-subject factor ‘rim/non-rim lesions’, random factor ‘patient’) was used to compare changes of logarithmically transformed relative volume fraction data. An unstructured covariance matrix was applied, because it showed the best fit to the data according to the Schwarz’s Bayesian Information Criterion (BIC). Other covariance matrices tested were Compound Symmetry, Autoregressiv 1, and a Diagonal Covariance Matrix. For modelling the random factor ‘patient’, multiple lesions per patient were considered. The reported *p* values are results of two-sided tests. *P* values ≤0.05 were considered to indicate significant results.

## Results

### Post-mortem study

#### Rim-like iron accumulation at edges of a subset of WM lesions

In the first sample of 28 MS cases with double-hemispheric or large sections, 183 WM MS lesions were found (Table 1). 7 lesions from 3 cases were active, 10 from 6 cases were active/inactive, 24 from 8 cases slowly expanding, 49 from 14 cases inactive, 19 from 7 cases partially remyelinated, and 74 from 15 cases were shadow plaques. Iron accumulation along some lesion edges formed a complete or incomplete rim (Fig. 1a, b). This was the case in 11 out of 24 slowly expanding lesions, defined by the presence of LFB-positive myelin degradation products in microglia/macrophages (Fig. 1c, inset). Slowly expanding lesions also showed elevated numbers of CD68-positive microglia/macrophages at the lesion edge (Fig. 1f, inset). Iron was mainly present in microglia/macrophages (Fig. 1i). Inactive edges were accompanied by the presence of lipofuscin-loaded macrophages (Fig. 1d, inset) on adjacent slides stained for LFB-PAS, indicating cessation of demyelinating activity months or years prior, while iron was still stored in the remaining cells. Density of CD68-positive microglia/macrophages was lower in inactive (Fig. 1g) than in slowly expanding edges (Fig. 1f). Edge-related iron accumulation in microglia/macrophages was observed around 6 out of 49 inactive lesions (Fig. 1j). The highest density of iron-laden microglia/macrophages found at the edge of an inactive lesion is shown in supplementary Fig. 1. Fully remyelinated lesions, so called shadow plaques, did not show conspicuous changes in CD68-positive cell densities, neither in the plaque edge nor in the center (Fig. 1h). Edge-related iron accumulation in microglia/macrophages was hardly observed around shadow plaques (Fig. 1k). The highest density of iron-laden microglia/macrophages found at a shadow plaque edge is displayed in supplementary Fig. 1. Active lesions did not show iron accumulation at the lesion edge (not shown). The majority of MS lesions, regardless of lesion type, displayed reduced overall iron load in the lesion center, when compared with the periplaque WM (Fig. 1).

#### Iron and microglia/macrophages in NAWM and WM lesions

Densities of CD68^+^ microglia/macrophages across regions of NAWM and different lesion subtypes confirmed proper region selection for cellular iron densities and countings (Fig. 2a, first sample). We observed significantly higher CD68 expression at the edges of active (Mann–Whitney *U* test, *p* = 0.003 after correction for multiple comparisons), active/inactive (*p* = 0.008), and slowly expanding lesions (*p* = 0.01), when compared with NAWM. Conversely, inactive edges did not significantly differ from NAWM in their level of CD68 expression (*p* = 1 after correction for multiple comparisons). Intracellular iron densities, determined by digital optical densitometry using the area fraction method in the ImageJ, were significantly different only at slowly expanding lesion edges compared with NAWM (Fig. 2b) (*p* = 0.01). Countings of iron-laden microglia/macrophages revealed a significant elevation of iron-laden microglia/macrophages both in slowly expanding (*p* = 0.045) and inactive lesion edges (*p* = 0.004), when compared with NAWM (Fig. 2c). No significant increase in edge-associated iron was seen in shadow plaques.

#### Activation status of iron-laden microglia/macrophages at slowly expanding lesion edges

The second sample of 10 MS cases harbored 10 lesions (1 lesion per case), all of which were classified as slowly expanding. These sections were double-labeled for iron and the pro-inflammatory marker p22phox [13], the costimulatory molecule CD86 (pro-inflammatory M1 activation), and CD206 (mannose receptor, anti-inflammatory M2 activation) [33] (Fig. 3). The majority of iron-laden microglia/macrophages at slowly expanding edges expressed CD86 and p22phox (Fig. 3a, b). However, microglia/macrophages devoid of iron also expressed these molecules in slowly expanding edges (Fig. 3d, e). Expression of CD206 at the lesion edge was generally sparse and predominantly found in perivascular macrophages devoid of iron (Fig. 3c). Of the few CD206-positive microglia/macrophages, a small minority was iron-laden, thus double-positive for CD206/iron (Fig. 3f). Manual countings of microglia/macrophages on sections stained for p22phox/CD206 with iron are displayed in supplementary Fig. 2. CD86 expression was very similar to p22phox expression at slowly expanding lesion edges; therefore, these stainings were not counted. In the whole second sample, iron-positive astrocytes were only exceptionally found in the iron rims and invariably surrounded by a majority of microglia/macrophages with a stronger iron staining (Fig. 3i, j). Six out of ten lesions of the second sample displayed focal perivascular iron accumulation in the lesion center (exemplified in Fig. 3h, black arrows). Iron-laden astrocytes were more numerous in these regions than in the iron rims (Fig. 3k, l).

#### Hypointensities at lesion rims reflect iron-laden microglia/macrophages in post mortem SWI

Post-mortem SWI disclosed four large periventricular MS lesions (one lesion per case), two of which are presented in Fig. 4. Two lesions were pathologically classified as slowly expanding and presented rims of decreased signal on SWI (Fig. 4a). These two lesions showed iron rims (Fig. 4b, f), which correlated with hyperintense phase (Fig. 4d), hypointense SWI (Fig. 4e), and iron-laden microglia/macrophages (Fig. 4g, h). In the other two lesions, which were pathologically classified as inactive, no rims of hypointense SWI (Fig. 4j, n) or hyperintense phase (Fig. 4m) were observed. Edge-related iron accumulation was absent (Fig. 4k, o), as were iron-laden microglia/macrophages (Fig. 4p). Iron-laden microglia/macrophages were counted along the edges of these 4 WM lesions in 54 ROIs, which were manually stratified into either matching with a dark SWI rim or not (Fig. 5a). ROIs matching with a dark SWI rim contained significantly more iron-laden microglia/macrophages (*p* < 0.001), providing a threshold of 47.5 iron-laden microglia/macrophages per image required to be reflected as a dark rim in post-mortem SWI. Applying this threshold to the countings in all 183 lesion edges of the first sample, we found a significant difference of the likelihood of slowly expanding lesions (11/24, 45.8%) versus inactive lesions (6/49, 12.2%) to exceed this threshold (Pearson Chi-square = 10.147; *p* = 0.003) (Fig. 5b). Two active/inactive, one out of seventy four shadow plaques, and none of the seven active or nineteen partially remyelinated lesions exceeded this threshold. The single shadow plaque edge exceeding the threshold is depicted in supplementary Fig. 1.

### In vivo study

The 7 T scan was well tolerated by all patients. Two patients complained of transient and short-lasting dizziness upon entering in the scanner. Of all examined MRIs obtained after contrast injection, i.e., at year 1, none showed enhancing lesions. Data of two patients with secondary progressive MS were excluded from the analysis due to movement artifacts. These two patients also could not complete the follow-up because of disability accretion. One patient with secondary progressive MS did not present any rim lesion and was not included in the lesion volume analysis (Patient 10 in Table 3). One patient missed the 3.5-year follow-up examination due to a newly implanted dental brace but was included in the analysis (Patient 1 in Table 3). Data from seven patients were thus included into longitudinal lesion volumetry.

#### In vivo incidence of WM lesions and evolution of rim lesions

At baseline, we counted 183 WM lesions on the FLAIR–SWI sequence. In addition, 14 lesions in 5 RRMS patients appeared newly within 3.5 years. None of these newly appearing lesions displayed rims and none were included in the longitudinal volumetric analysis. Of the 183 WM lesions present at baseline, 28 (15.3%) were surrounded by a rim of decreased signal on SWI, while 24 (13.2%) were discrete and did not show a rim. The remaining 131 lesions (71.5%) did not show a rim and were confluent, spanning over large areas of the brains. Thus, 84.5% of the 155 non-rim lesions were confluent and not included in the volumetric analysis. 52 lesions (28 rim lesions, 24 discrete non-rim lesions) were included in the volumetric analysis. Since one patient missed the last follow-up, 47 lesions (26 rim lesions, 21 discrete non-rim lesions) were analyzed at last follow-up.

**Fig. 6:**
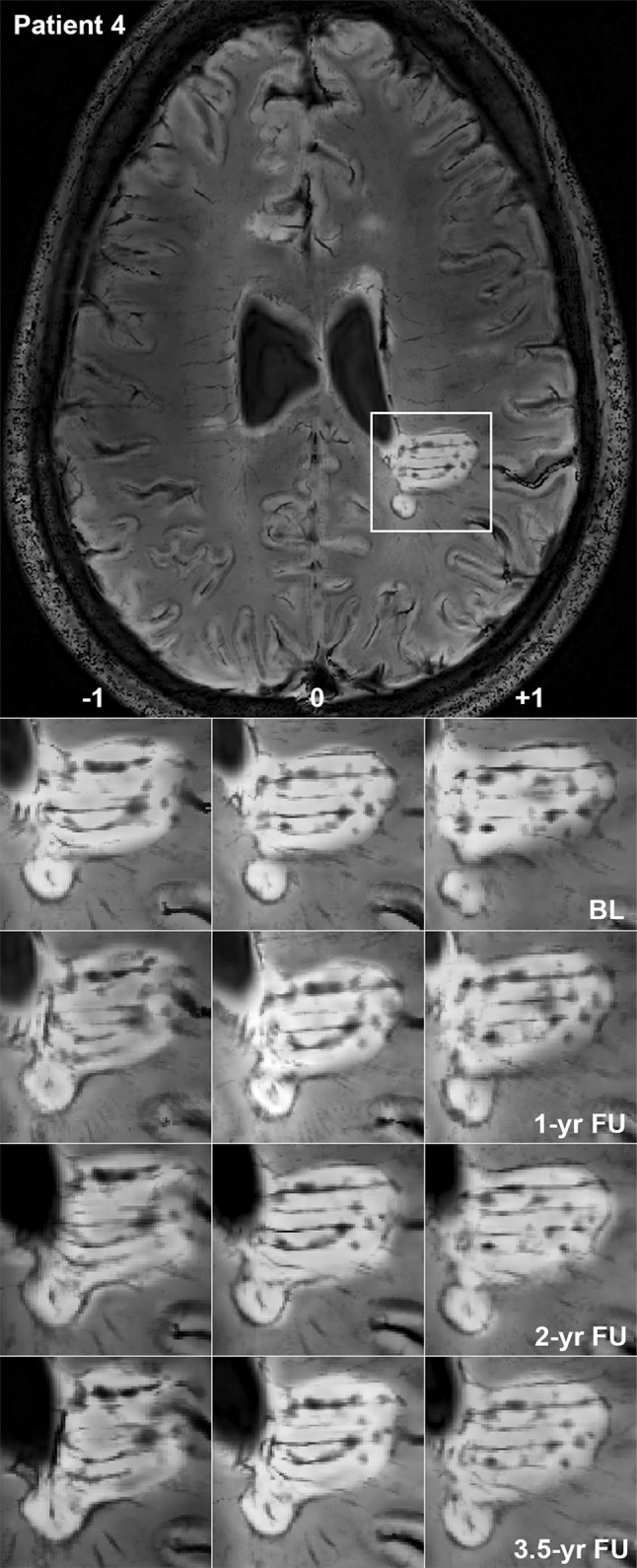
Expansion of a rim lesion. Patient 4, a 29-year-old lady with relapsing-remitting multiple sclerosis lasting for 5 years, EDSS 2. 7 T FLAIR–SWI data show several WM hyperintense lesions typical for the disease. One large periventricular hyperintense lesion with an encircling hypointense rim is indicated by a *white rectangle* and magnified. Within this lesion, tubular hypointense structures suggestive of veins and circumscribed nodular hypointensities are visible. Images demonstrate a slow expansion of the posterior parts of this lesion over 3.5 years, leading to a fusion of initially separated lesion parts. Three contiguous imaging slices (−1, 0, +1) show that expansion and fusion are not due to willful slice sampling. Note the global brain atrophy of this patient over time, which is evident from the widening of the ventricles. *BL* baseline, *FU* follow-up, *yr* year

**Fig. 7:**
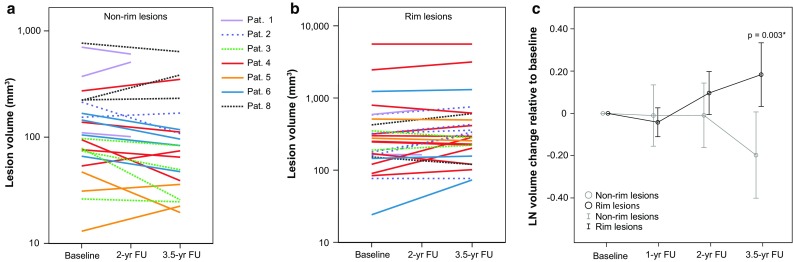
Volumetric longitudinal data of non-rim and rim lesions. Absolute lesional volumes of non-rim (**a**) and rim lesions (**b**) in mm^3^. Data points from baseline and last available follow-up of single lesion volumes are plotted on a logarithmic scale. **c** Logarithmically transformed lesion volume changes relative to baseline. Mean values (*circles*) are plotted with 95% confidence intervals (*bars*). After 3.5 years, rim lesion volumes showed significant expansion over time compared with non-rim lesions that on average shrink. *P value indicates significantly different volume developments between rim and non-rim lesions (mixed-model ANOVA, factor ‘rim*timepoint’). *FU* follow-up, *yr* year

Rim lesions were observed in six RRMS and one SPMS patient (Table 3). Neither appearance nor disappearance of rims was noted during the study period, i.e., all of the observed rims were persistent. In two patients, parts of two rim lesions expanded over time and coalesced into one large lesion. Expansion of the posterior parts of such a rim lesion together with fusion of initially separated lesion parts is shown in Fig. 6. Rim lesions were significantly larger than discrete non-rim lesions at each timepoint (Mann–Whitney *U* tests; *p* = 0.019 at baseline, *p* = 0.003 at 1- and 2-year follow-up, *p* < 0.001 at 3.5-year follow-up). Single lesion volumes with data points connected from baseline and last available follow-up are displayed in Fig. 7a (non-rim lesions) and b (rim lesions). Over 3.5 years, rim lesions expanded on average by 29.33%, whereas non-rim lesions decreased by 10.04%. The volume developments over time were significantly different between rim and non-rim lesions (mixed-model ANOVA, factor ‘iron rim*timepoint’, F_49.552_ = 6.746, *p* = 0.003) (Fig. 7c), also when accounting for patients as random factor in the model. Intraclass correlation of 15 lesion volumes measured twice was *r* = 0.998.

## Discussion

We report four key observations in our present study: (1) An iron rim at the edge of an MS lesion is predominantly seen in slowly expanding lesions, much less frequently in inactive lesions, not in active and hardly in remyelinated lesions. (2) The iron containing cells in the rim are in their vast majority microglia/macrophages with a pro-inflammatory activation status, while iron-positive astrocytes are sparse or absent. (3) Direct 7 T MRI—pathology correlation shows that the iron rim, defined by pathology, can be reliably visualized by magnetic resonance imaging. (4) Lesions with an iron rim on average expand very slowly, while non-rim lesions show a tendency to shrink. Although expansion of rim lesions has already been observed in individual MS lesions [2, 3], we have statistically proven this expansion for the first time in multiple MS patients and lesions. However, it is important to note that some non-rim lesions expanded and some rim lesions shrinked. Our data, therefore, indicate that an observed rim lesion does not necessarily expand over time, but has a higher probability to do so, when compared with non-rim lesions. Using post-mortem pathology-imaging correlations, we confirm this rim to be due to the presence of iron inside pro-inflammatory activated microglia/macrophages [3, 5, 22]. In line with pathological [5, 21, 30, 34] and in vivo [3, 39, 40] studies, this pattern of iron accumulation was restricted to edges of chronically demyelinated lesions, not present in active lesions [1], and hardly observed in fully remyelinated shadow plaques [21].

Pathologically, we observed the most pronounced iron accumulation at the rims of slowly expanding lesions. The empirically derived threshold for iron-laden microglia/macrophages sufficient to decrease the signal in post-mortem SWI revealed a significant association of slowly expanding lesions with a rim in SWI, when compared with inactive lesions. However, translating this threshold to the in vivo situation needs to be exerted with caution, given the differences in imaging resolution and tissue properties between in vivo and post-mortem imaging. One explanation for the absence of iron rims in classical active lesions could be the dynamics of lesion formation. In active lesions, myelin fragments are mainly taken up by cells with a macrophage phenotype (round, no processes). These are mobile, disperse within the lesion, and finally accumulate in perivascular spaces. Conversely, in slowly expanding lesions, tissue debris is taken up by cells with microglia phenotype (branched cells with processes). Microglia may remain stationary at lesion edges for prolonged time periods, which may form the basis for the persistent iron rim. Lack of iron accumulation in the vast majority of astrocytes found in iron rims could be related to the elevated expression of the iron exporter ferroxidase ceruloplasmin in astrocytes at edges of MS lesions [21], indicating active iron efflux by astrocytes.

Microglia and macrophages in iron rims highly expressed the pro-inflammatory markers CD86 and p22phox, while anti-inflammatory CD206 (mannose receptor) expression was rare and, in line with prior results [38], mainly expressed by perivascular macrophages. In the mentioned study [38], CD206 in active MS lesions was also expressed by 70% of myelin-laden foamy macrophages which expressed M1 markers. Thus, the authors proposed an intermediate activation status of the majority of macrophages in active MS lesions. This situation is different for edges of slowly expanding lesions, as reported here, where the majority of microglia/macrophages show a clear pro-inflammatory activation status without CD206 expression. A similar concept has been proposed by Pitt and collaborators [30], who also showed that CD206 was mainly expressed by lipid-laden macrophages in MS lesions, while iron-laden microglia/macrophages at edges of lesions did not express CD206. We have additionally shown the expression of pro- and anti-inflammatory markers in double-labelings with iron in a sample of ten well-characterized slowly expanding lesions. Expression of pro-inflammatory markers in microglia/macrophages was independent from iron accumulation in these cells in our study. Therefore, the iron rim indicates microglia/macrophages with a pro-inflammatory activation status, but iron itself does not seem to induce pro-inflammatory activation, as determined by the markers we have included (CD86, p22phox). Our interpretation is that pro-inflammatory microglia/macrophages at edges of slowly expanding lesions either accumulate iron or not, but iron rims specifically indicate microglia/macrophages with a pro-inflammatory activation status.

Lack of iron rims around the vast majority of shadow plaques is particularly informative in comparison with inactive lesions. This feature might indicate that remyelination is restricted to lesions which have never undergone a stage of edge iron accumulation, because otherwise traces of long-standing edge iron in a subset of remyelinated shadow plaques would be expected. However, extended follow-up studies of rim lesions will be able to investigate whether they do not remyelinate.

Our data are in line with another study showing lack of remyelination in five lesions of a single progressive MS autopsy case displaying phase rims and pathologically confirmed iron accumulation [3]. Based on in vivo data, the authors conclude from a progressively lower *T*1 intensity between 3 and 12 months of observation seen in 7/10 lesions with persistent phase rim versus 7/26 lesions without phase rim that the phase rim lesions showed failure of early tissue repair and possibly remyelination failure, as opposed to non-rim lesions. This is in line with our observation in 74 shadow plaques of 15 MS autopsy cases, showing the absence of remyelination in iron rim lesions and the absence of iron rims in remyelinated lesions. While *T*2-weighted or FLAIR images are unable to separate remyelinated from demyelinated lesions [6], SWI could, therefore, help to distinguish them.

We do not think that remyelination might explain the observed shrinking of hyperintense non-rim lesions in our survey, since remyelinated lesions were as *T*2-hyperintense as completely demyelinated lesions in a post-mortem correlation study [6]. Conversely, we ascribe the -10% baseline volume of non-rim lesions to lesion-specific gliosis and neurodegeneration, which leads to tissue retraction and shrinkage [28].

The frequency of rims around WM lesions in our in vivo data (15.3% of all observed WM lesions) is similar to that of another in vivo study, where authors reported 10.1% of all MS WM lesions to display a rim indicative of iron accumulation [10]. These authors furthermore noted that the majority (83.3%) of MS lesions lacking MRI signs suggestive of iron accumulation were ill-defined and showed faint margins. In comparison, 84.5% of our non-rim lesions were ill-defined and confluent and, therefore, not included in volumetric analysis, which is crucially dependent on a reliable depiction of the lesion margin.

Rim lesions were significantly larger than non-rim lesions at each timepoint, which is in line with other data [3]. First, larger size of rim lesions could be the result of their expansion together with shrinkage of non-rim lesions over time. Second, lesions which are larger at baseline could be more likely to end up as slowly expanding. The latter possibility is suggested by data from Absinta et al. [3]. In this study, newly forming and enhancing lesions, which later developed a persistent phase rim, were already larger at baseline (timepoint of gadolinium enhancement) than lesions which did not develop a persistent phase rim.

## Limitations of the study

The main limitation of this study arises from the small cohort of MS patients and low number of lesions which were manually traced for volumetry in vivo. Future studies on larger patient cohorts are, therefore, needed for the relation between presence of rim lesions and, ultimately, MS disease course or severity. Another limitation comes from the fact that MS cases, sequence parameters and tissue conditions were not identical between in vivo and post-mortem imaging. Therefore, the translation of post-mortem and in vivo MRI findings needs to be perceived with caution. Furthermore, we could not account for the effect of whole brain atrophy on longitudinal lesion volumes in this study, since brain volumetry using 7 T MRI is still challenging [37].

## Conclusions

Our study shows that iron in the brains of patients with relapsing or progressive MS accumulates at the edge of slowly expanding lesions in microglia/macrophages. There it persists for months to years when lesions are slowly expanding or when demyelinating activity has ceased. This iron deposition is visible by susceptibility-based MRI and indicates chronic lesions with pro-inflammatory microglia/macrophages, complete myelin loss within the rim and absence of remyelination. On average, MS lesions with iron rims expand slowly, while lesions without iron rims show a tendency to decrease in volume. In remyelinated lesions, iron rims have hardly been observed, indicating that they may impair myelin repair. These data suggest that the presence of iron rims in MRI images of MS brains may be a sign of progressive tissue injury. Whether iron rims may become a paraclinical marker for disease activity or prognosis in patients with relapsing and progressive MS, has to be determined in future prospective studies.

## Electronic supplementary material

Below is the link to the electronic supplementary material.

Supplementary material 1 (DOCX 809 kb)

Supplementary material 2 (DOCX 159 kb)

## References

[1] Absinta M, Sati P, Gaitan MI, Maggi P, Cortese IC, Filippi M, Reich DS (2013). Seven-tesla phase imaging of acute multiple sclerosis lesions: a new window into the inflammatory process. Ann Neurol.

[2] Absinta M, Sati P, Reich DS (2016). Advanced MRI and staging of multiple sclerosis lesions. Nature Rev Neurol.

[3] Absinta M, Sati P, Schindler M, Leibovitch EC, Ohayon J, Wu T, Meani A, Filippi M, Jacobson S, Cortese IC et al (2016) Persistent 7-tesla phase rim predicts poor outcome in new multiple sclerosis patient lesions. J Clin Investig. doi:10.1172/JCI86198 10.1172/JCI86198 PMC4922708 27270171

[4] Babbe H, Roers A, Waisman A, Lassmann H, Goebels N, Hohlfeld R, Friese M, Schroder R, Deckert M, Schmidt S (2000). Clonal expansions of CD8(+) T cells dominate the T cell infiltrate in active multiple sclerosis lesions as shown by micromanipulation and single cell polymerase chain reaction. J Exp Med.

[5] Bagnato F, Hametner S, Yao B, van Gelderen P, Merkle H, Cantor FK, Lassmann H, Duyn JH (2011). Tracking iron in multiple sclerosis: a combined imaging and histopathological study at 7 Tesla. Brain: J Neurol.

[6] Barkhof F, Bruck W, De Groot CJ, Bergers E, Hulshof S, Geurts J, Polman CH, van der Valk P (2003). Remyelinated lesions in multiple sclerosis: magnetic resonance image appearance. Arch Neurol.

[7] Bian W, Harter K, Hammond-Rosenbluth KE, Lupo JM, Xu D, Kelley DA, Vigneron DB, Nelson SJ, Pelletier D (2013). A serial in vivo 7 T magnetic resonance phase imaging study of white matter lesions in multiple sclerosis. Mult Scler.

[8] Bramow S, Frischer JM, Lassmann H, Koch-Henriksen N, Lucchinetti CF, Sorensen PS, Laursen H (2010). Demyelination versus remyelination in progressive multiple sclerosis. Brain: J Neurol.

[9] Bruck W, Porada P, Poser S, Rieckmann P, Hanefeld F, Kretzschmar HA, Lassmann H (1995). Monocyte/macrophage differentiation in early multiple sclerosis lesions. Ann Neurol.

[10] Chawla S, Kister I, Wuerfel J, Brisset JC, Liu S, Sinnecker T, Dusek P, Haacke EM, Paul F, Ge Y (2016) Iron and non-iron-related characteristics of multiple sclerosis and neuromyelitis optica lesions at 7 T MRI. AJNR Am J Neuroradiol. doi:10.3174/ajnr.A4729 10.3174/ajnr.A4729 PMC4946971 27012298

[11] Dal-Bianco A, Hametner S, Grabner G, Schernthaner M, Kronnerwetter C, Reitner A, Vass C, Kircher K, Auff E, Leutmezer F (2015). Veins in plaques of multiple sclerosis patients—a longitudinal magnetic resonance imaging study at 7 Tesla. Eur Radiol.

[12] Eskreis-Winkler S, Deh K, Gupta A, Liu T, Wisnieff C, Jin M, Gauthier SA, Wang Y, Spincemaille P (2015). Multiple sclerosis lesion geometry in quantitative susceptibility mapping (QSM) and phase imaging. J Magn Reson Imaging: JMRI.

[13] Fischer MT, Sharma R, Lim JL, Haider L, Frischer JM, Drexhage J, Mahad D, Bradl M, van Horssen J, Lassmann H (2012). NADPH oxidase expression in active multiple sclerosis lesions in relation to oxidative tissue damage and mitochondrial injury. Brain: J Neurol.

[14] Frischer JM, Bramow S, Dal-Bianco A, Lucchinetti CF, Rauschka H, Schmidbauer M, Laursen H, Sorensen PS, Lassmann H (2009). The relation between inflammation and neurodegeneration in multiple sclerosis brains. Brain: J Neurol.

[15] Frischer JM, Weigand SD, Guo Y, Kale N, Parisi JE, Pirko I, Mandrekar J, Bramow S, Metz I, Bruck W (2015). Clinical and pathological insights into the dynamic nature of the white matter multiple sclerosis plaque. Ann Neurol.

[16] Goldschmidt T, Antel J, Konig FB, Bruck W, Kuhlmann T (2009). Remyelination capacity of the MS brain decreases with disease chronicity. Neurology.

[17] Grabner G, Dal-Bianco A, Schernthaner M, Vass K, Lassmann H, Trattnig S (2011). Analysis of multiple sclerosis lesions using a fusion of 3.0 T FLAIR and 7.0 T SWI phase: FLAIR SWI. J Magn Reson Imaging: JMRI.

[18] Haacke EM, Makki M, Ge Y, Maheshwari M, Sehgal V, Hu J, Selvan M, Wu Z, Latif Z, Xuan Y (2009). Characterizing iron deposition in multiple sclerosis lesions using susceptibility weighted imaging. J Magn Reson Imaging: JMRI.

[19] Haacke EM, Xu Y, Cheng YC, Reichenbach JR (2004). Susceptibility weighted imaging (SWI). Magn Reson Med.

[20] Haider L, Fischer MT, Frischer JM, Bauer J, Hoftberger R, Botond G, Esterbauer H, Binder CJ, Witztum JL, Lassmann H (2011). Oxidative damage in multiple sclerosis lesions. Brain: J Neurol.

[21] Hametner S, Wimmer I, Haider L, Pfeifenbring S, Bruck W, Lassmann H (2013). Iron and neurodegeneration in the multiple sclerosis brain. Ann Neurol.

[22] Hammond KE, Metcalf M, Carvajal L, Okuda DT, Srinivasan R, Vigneron D, Nelson SJ, Pelletier D (2008). Quantitative in vivo magnetic resonance imaging of multiple sclerosis at 7 Tesla with sensitivity to iron. Ann Neurol.

[23] Irvine KA, Blakemore WF (2008). Remyelination protects axons from demyelination-associated axon degeneration. Brain: J Neurol.

[24] Kornek B, Storch MK, Weissert R, Wallstroem E, Stefferl A, Olsson T, Linington C, Schmidbauer M, Lassmann H (2000). Multiple sclerosis and chronic autoimmune encephalomyelitis: a comparative quantitative study of axonal injury in active, inactive, and remyelinated lesions. Am J Pathol.

[25] Kurtzke JF (1983). Rating neurologic impairment in multiple sclerosis: an expanded disability status scale (EDSS). Neurology.

[26] Kutzelnigg A, Lucchinetti CF, Stadelmann C, Bruck W, Rauschka H, Bergmann M, Schmidbauer M, Parisi JE, Lassmann H (2005). Cortical demyelination and diffuse white matter injury in multiple sclerosis. Brain: J Neurol.

[27] Lassmann H (2011). Review: the architecture of inflammatory demyelinating lesions: implications for studies on pathogenesis. Neuropathol Appl Neurobiol.

[28] Mahad DH, Trapp BD, Lassmann H (2015). Pathological mechanisms in progressive multiple sclerosis. Lancet Neurol.

[29] Meguro R, Asano Y, Odagiri S, Li C, Iwatsuki H, Shoumura K (2007). Nonheme-iron histochemistry for light and electron microscopy: a historical, theoretical and technical review. Arch Histol Cytol.

[30] Mehta V, Pei W, Yang G, Li S, Swamy E, Boster A, Schmalbrock P, Pitt D (2013). Iron is a sensitive biomarker for inflammation in multiple sclerosis lesions. PLoS One.

[31] Noll DC, Nishimura DG, Macovski A (1991). Homodyne detection in magnetic resonance imaging. IEEE Trans Med Imaging.

[32] Ontaneda D, Fox RJ (2015). Progressive multiple sclerosis. Curr Opin Neurol.

[33] Peferoen LA, Vogel DY, Ummenthum K, Breur M, Heijnen PD, Gerritsen WH, Peferoen-Baert RM, van der Valk P, Dijkstra CD, Amor S (2015). Activation status of human microglia is dependent on lesion formation stage and remyelination in multiple sclerosis. J Neuropathol Exp Neurol.

[34] Pitt D, Boster A, Pei W, Wohleb E, Jasne A, Zachariah CR, Rammohan K, Knopp MV, Schmalbrock P (2010). Imaging cortical lesions in multiple sclerosis with ultra-high-field magnetic resonance imaging. Arch Neurol.

[35] Polman CH, Reingold SC, Edan G, Filippi M, Hartung HP, Kappos L, Lublin FD, Metz LM, McFarland HF, O’Connor PW (2005). Diagnostic criteria for multiple sclerosis: 2005 revisions to the “McDonald Criteria”. Ann Neurol.

[36] Prineas JW, Kwon EE, Cho ES, Sharer LR, Barnett MH, Oleszak EL, Hoffman B, Morgan BP (2001). Immunopathology of secondary-progressive multiple sclerosis. Ann Neurol.

[37] Van de Moortele PF, Auerbach EJ, Olman C, Yacoub E, Ugurbil K, Moeller S (2009). T1 weighted brain images at 7 Tesla unbiased for Proton Density, *T*2* contrast and RF coil receive B1 sensitivity with simultaneous vessel visualization. NeuroImage.

[38] Vogel DY, Vereyken EJ, Glim JE, Heijnen PD, Moeton M, van der Valk P, Amor S, Teunissen CE, van Horssen J, Dijkstra CD (2013). Macrophages in inflammatory multiple sclerosis lesions have an intermediate activation status. J Neuroinflammation.

[39] Yao B, Bagnato F, Matsuura E, Merkle H, van Gelderen P, Cantor FK, Duyn JH (2012). Chronic multiple sclerosis lesions: characterization with high-field-strength MR imaging. Radiology.

[40] Yao B, Ikonomidou VN, Cantor FK, Ohayon JM, Duyn J, Bagnato F (2015). Heterogeneity of Multiple Sclerosis White Matter Lesions Detected With *T*2*-Weighted Imaging at 7.0 Tesla. J Neuroimaging: Off J Am Soc Neuroimaging.

