# Supplementary material for: Slow expansion of multiple sclerosis iron rim lesions: pathology and 7 T magnetic resonance imaging
Source: Acta Neuropathol. 2016 Oct 27;133(1):25–42. doi: 10.1007/s00401-016-1636-z (PMC5209400; doi:10.1007/s00401-016-1636-z)
Supplement: Supplementary file 1 — Supplementary material 1 (DOCX 809 kb) [file 401_2016_1636_MOESM1_ESM.docx]

Supplementary figure 1:


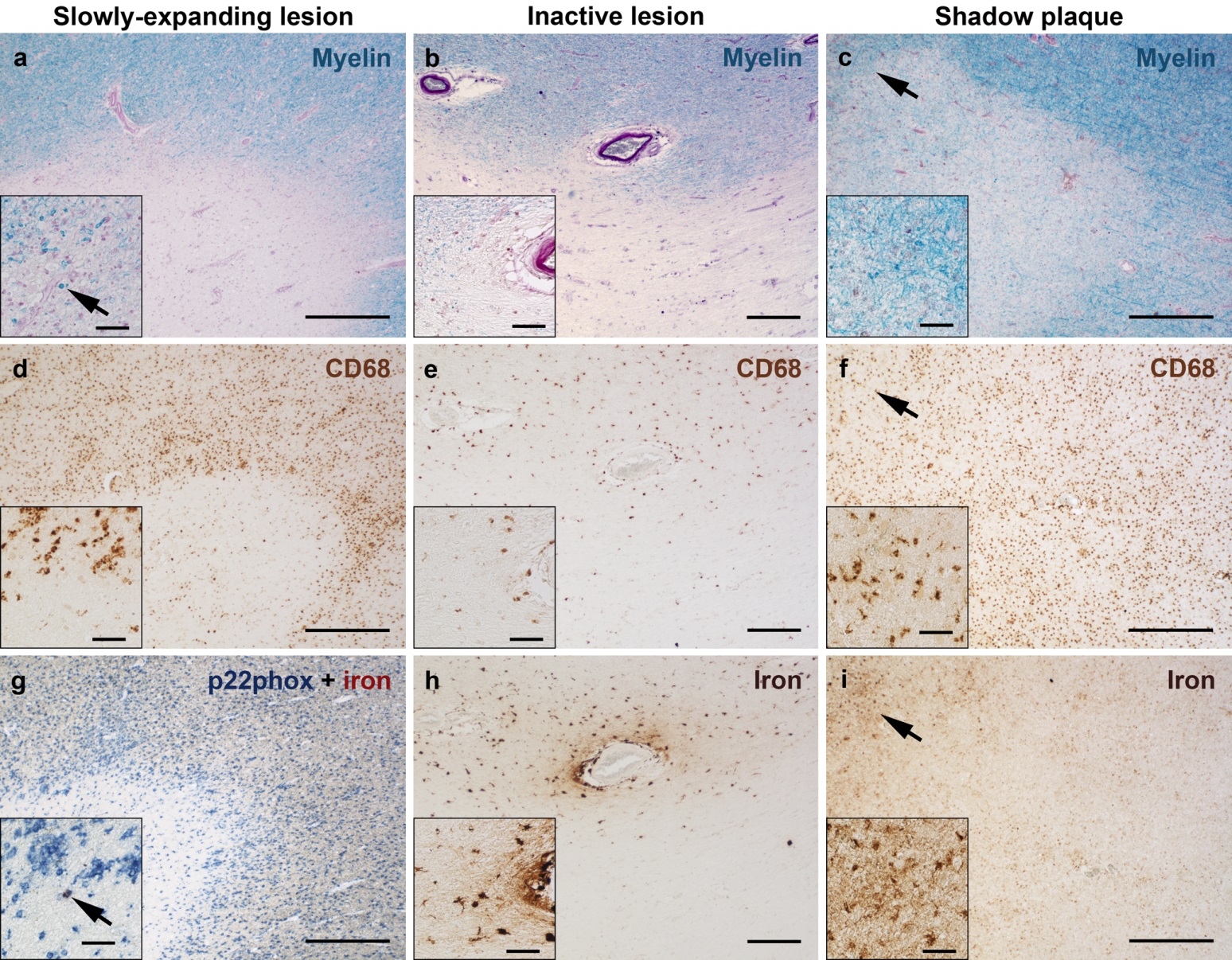


**Supplementary figure 1** Additional examples of absence or presence of lesion-edge related iron accumulation. (**a**, **d**, **g**) Slowly expanding lesion with LFB-positive myelin degradation products at the edge (arrow in inset **a**), elevated expression of CD68 (**d**), and only sporadic iron-laden microglia / macrophages (arrow in inset **g**). Despite the nearly complete absence of iron-laden microglia / macrophages, there is high expression of the pro-inflammatory marker p22phox of microglia / macrophages at the lesion edge (**g**). (**b**, **e**, **h**) Inactive lesion without degradation products at the edge (**b**), low expression of CD68 (**e**) and presence of iron-laden microglia / macrophages (**h**). This region shows the highest density of edge-related iron accumulation in microglia / macrophages in our whole sample of 49 inactive lesions. (**c**, **f**, **i**) Shadow plaque with sharply demarcated LFB myelin staining intensity reduction (**c**), unchanged CD68 expression across the periplaque WM, plaque borders and core (**f**), and iron accumulation in microglia / macrophages confined to an area close to the plaque border (indicated by arrows and magnified by insets in **c**, **f**, **i**). This region shows the highest density of iron-laden microglia / macrophages located at a shadow plaque edge in our whole sample of 74 shadow plaques. The region depicted in the inset of (**i**) corresponds to the data point of the single shadow plaque edge which exceeded the threshold in Figure 5

Scale bars = 500 µm (**a**, **d**, **g**, **c**, **f**, **i**); 200 µm (**b**, **e**, **h**); inset scale bars = 20 µm (**a**); 50 µm (all other insets)
