# Supplementary material for: Slow expansion of multiple sclerosis iron rim lesions: pathology and 7 T magnetic resonance imaging
Source: Acta Neuropathol. 2016 Oct 27;133(1):25–42. doi: 10.1007/s00401-016-1636-z (PMC5209400; doi:10.1007/s00401-016-1636-z)
Supplement: Supplementary file 2 — Supplementary material 2 (DOCX 159 kb) [file 401_2016_1636_MOESM2_ESM.docx]

Supplementary figure 2:


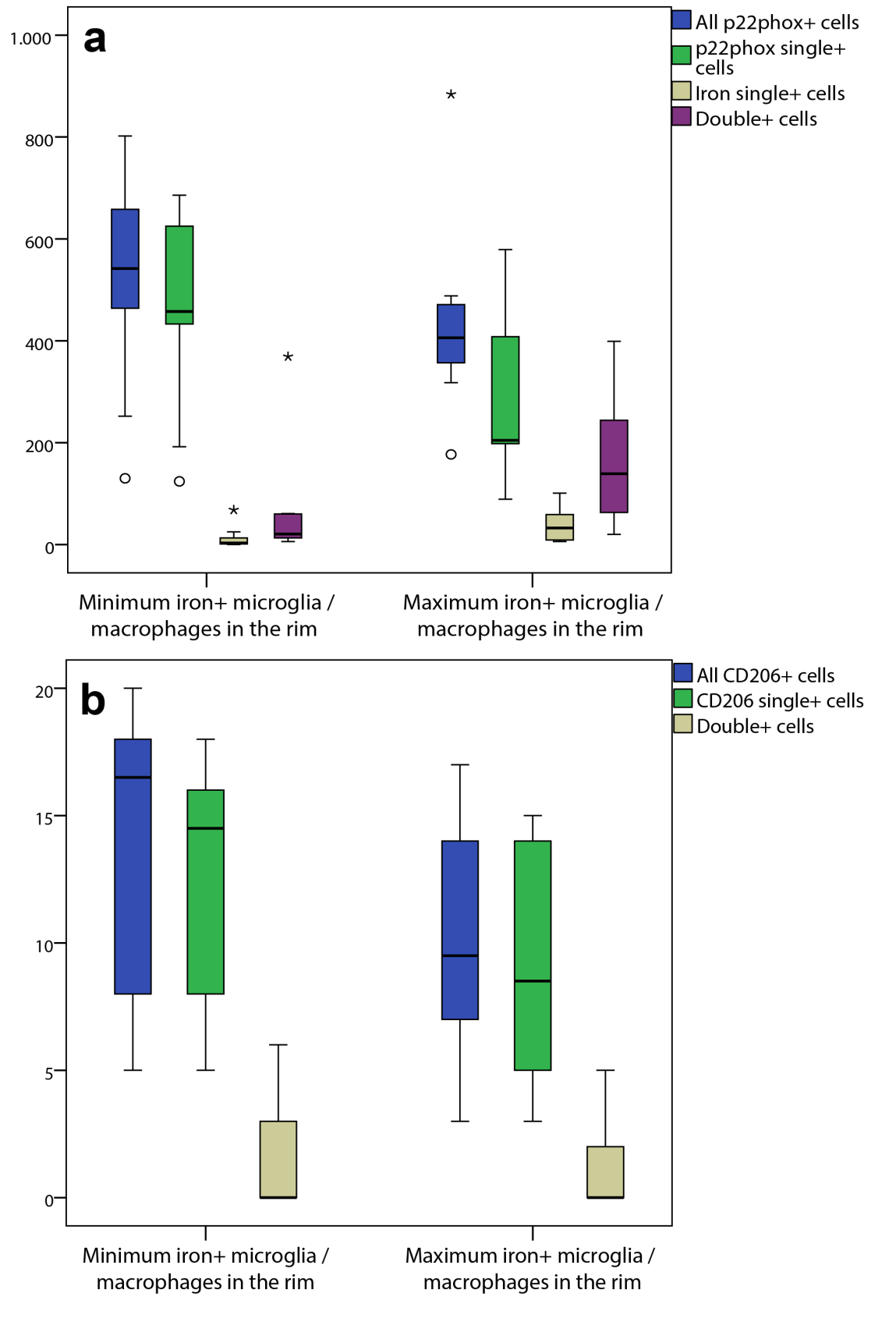


**Supplementary figure 2** Expression of pro-inflammatory p22phox (**a**) and anti-inflammatory CD206 (**b**) in microglia / macrophages at edges of 10 slowly expanding lesions of 10 MS cases. For each lesion edge, cell numbers / 0.75 mm^2^ were counted in 2 different ROIs: one with a minimum and one with a maximum of iron-laden cells in the edge. Expression of p22phox (**a**) was generally high at slowly expanding lesion edges (regardless of numbers of iron-laden cells), while expression of CD206 (**b**) was generally low
